# Spatial Temporal Dynamics and Molecular Evolution of Re-Emerging Rabies Virus in Taiwan

**DOI:** 10.3390/ijms17030392

**Published:** 2016-03-17

**Authors:** Yung-Cheng Lin, Pei-Yu Chu, Mei-Yin Chang, Kuang-Liang Hsiao, Jih-Hui Lin, Hsin-Fu Liu

**Affiliations:** 1 Department of Bioscience and Biotechnology, National Taiwan Ocean University, Keelung 20224, Taiwan; lin65mt@gmail.com (Y.-C.L.); gainx.bahamut@gmail.com (K.-L.H.); 2 Department of Medical Research, Mackay Memorial Hospital, Taipei 10449, Taiwan; 3 Department of Medical Laboratory Science and Biotechnology, Kaohsiung Medical University, Kaohsiung 80708, Taiwan; peiyuchu@kmu.edu.tw; 4 Department of Medical Laboratory Science and Biotechnology, Fooyin University, Kaohsiung 83102, Taiwan; cmy7328@ms37.hinet.net; 5 Center for Diagnostics and Vaccine Development, Centers for Disease Control, Taipei 11561, Taiwan; 6 Department of Nursing, National Taipei University of Nursing and Health Sciences, Taipei 11219, Taiwan

**Keywords:** rabies virus, phylogeographic, Formosan ferret badgers

## Abstract

Taiwan has been recognized by the World Organization for Animal Health as rabies-free since 1961. Surprisingly, rabies virus (RABV) was identified in a dead Formosan ferret badger in July 2013. Later, more infected ferret badgers were reported from different geographic regions of Taiwan. In order to know its evolutionary history and spatial temporal dynamics of this virus, phylogeny was reconstructed by maximum likelihood and Bayesian methods based on the full-length of glycoprotein (G), matrix protein (M), and nucleoprotein (N) genes. The evolutionary rates and phylogeographic were determined using Beast and SPREAD software. Phylogenetic trees showed a monophyletic group containing all of RABV isolates from Taiwan and it further separated into three sub-groups. The estimated nucleotide substitution rates of G, M, and N genes were between 2.49 × 10^−4^–4.75 × 10^−4^ substitutions/site/year, and the mean ratio of dN/dS was significantly low. The time of the most recent common ancestor was estimated around 75, 89, and 170 years, respectively. Phylogeographic analysis suggested the origin of the epidemic could be in Eastern Taiwan, then the Formosan ferret badger moved across the Central Range of Taiwan to western regions and separated into two branches. In this study, we illustrated the evolution history and phylogeographic of RABV in Formosan ferret badgers.

## 1. Introduction

Rabies virus (RABV) is the etiological agent of rabies which is a lethal zoonotic infection in the world, especially in Africa and Asia [1,2] of which human mortality was estimated to be 55,000 deaths per year [1]. The main RABV reservoirs are dogs and bats; nevertheless, dogs are the main transmitter of rabies cases [3,4,5]. RABV belongs to the genus *Lyssavirus*, family Rhabdoviridae*,* and is a single-stranded, negative-sense RNA virus with a genome size of approximately 12 kb which encodes five proteins: nucleoprotein (N), phosphoprotein (P), matrix protein (M), glycoprotein (G), and large protein (L) [6]. Fourteen species of *Lyssavirus* have been determined by phylogenetic analysis [2,7], and RABV is in the *Lyssavirus* phylogroup 1 [2,7,8,9]. These RNA viruses infect mammalian hosts via saliva by biting or licking, then transport and replicate in the central nervous system resulting in the formation of Negri body and host behavioral alterations [9].

The World Organization for Animal Health (OIE) has recognized Taiwan as a rabies-free country since 1961. However, this situation changed in 2013 by the discovery of rabies virus infected Formosan ferret badgers (*Melogale moschata subaurantiaca*) [10,11]. The Formosan ferret badger is an endemic subspecies in Taiwan. Its major habitat was distributed in the plains to 2000 meters of secondary forest bushes, such as the Coast Mountains in Eastern Taiwan. They are fierce and agile nocturnal carnivores, hunting small rodents or birds. Since the first rabies-infected animal was confirmed on 16 July 2013, a total of 1646 wild carnivores, 3231 dogs, 145 cats, 279 bats, and 404 other wild animals have been examined up until 31 December 2015. Almost all the rabies-infected animals were Formosan ferret badgers (511 cases). The other rabies positive cases were six masked palm civets, one dog, and one Asian house shrew. An important clue to understanding the reemergence of RABV in Taiwan is the branching time of Taiwan lineage and its evolutionary, spatial and temporal dynamics. For this purpose, we conduct a series of evolutionary studies to characterize the molecular phylogeny and phylogeography of the re-emerge rabies virus in Taiwan.

## 2. Results

### 2.1. Phylogenetic and Molecular Evolution of the Rabies Virus

**Figure 1:**
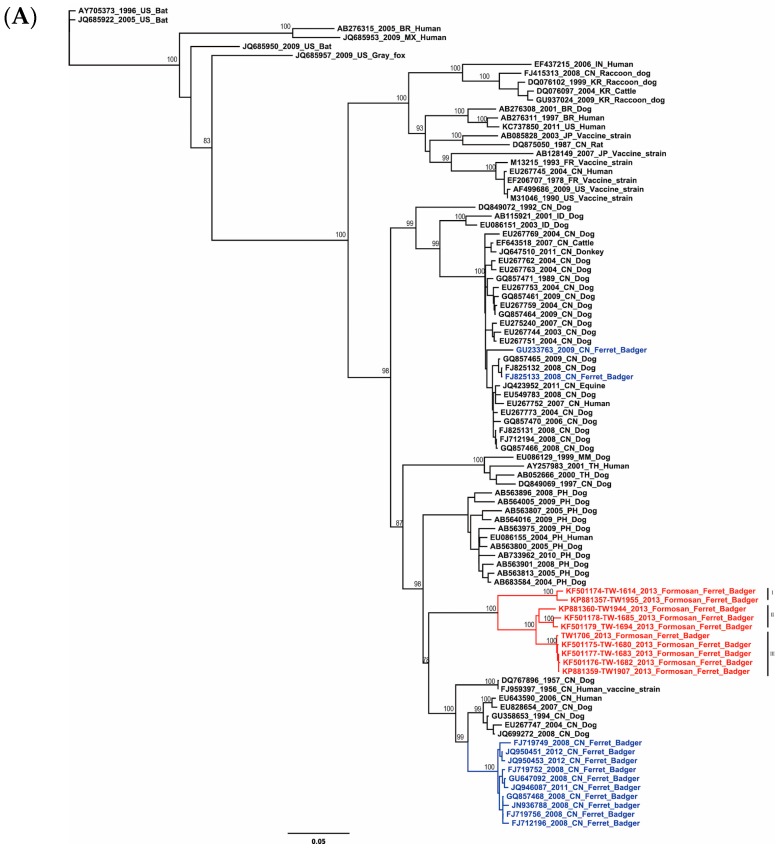

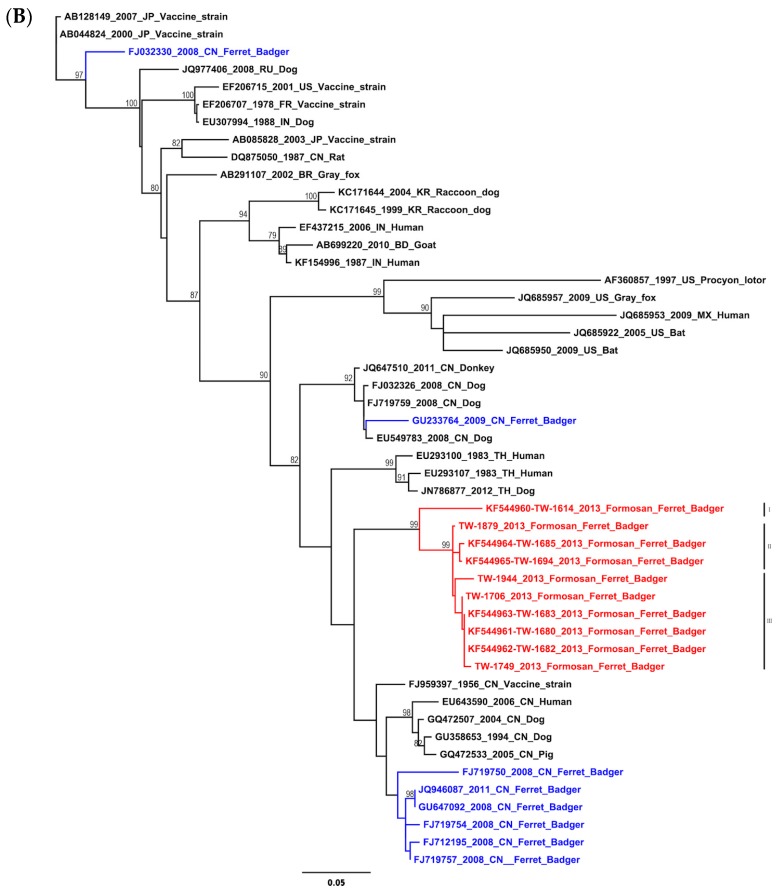

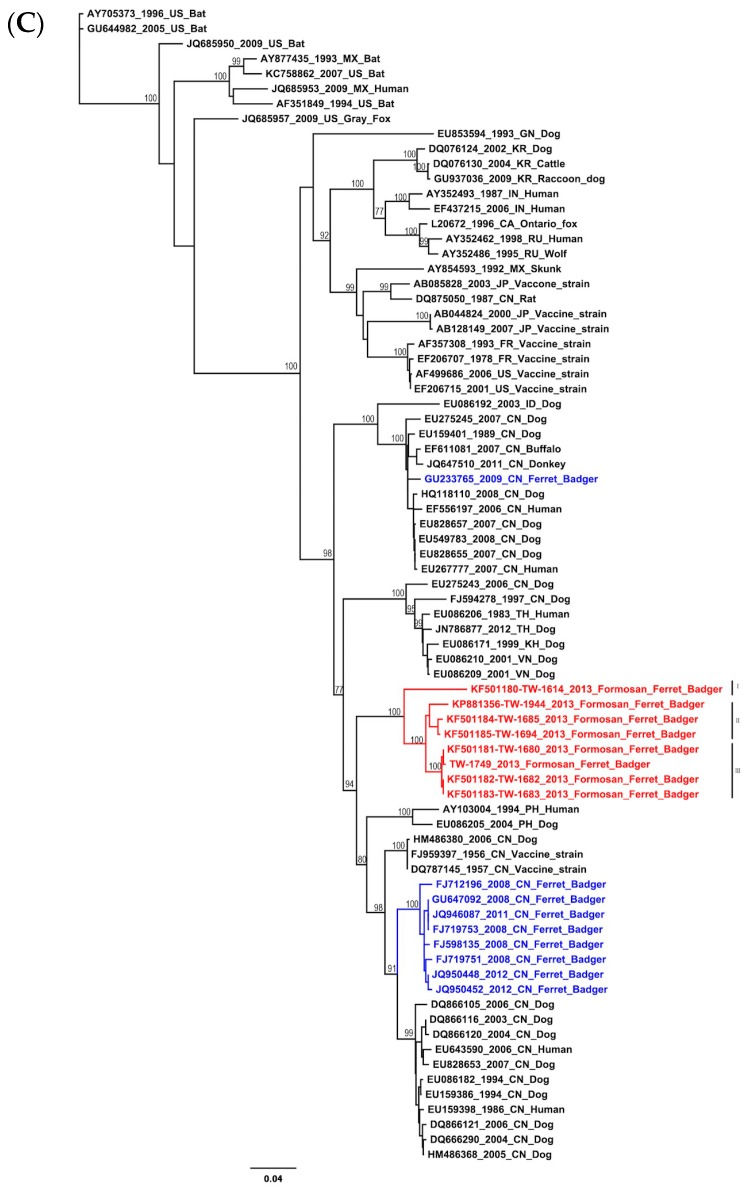
Phylogenetic trees of the rabies virus based on (**A**) glycolprotein, (**B**) matrix, and (**C**) nucleoprotein genes. Tree topology was constructed by the maximum likelihood method. Numbers beside the branches are bootstrap values as percentages (1000 replicates). Texts in red and blue represent the RABV isolated from Formosan and Chinese ferret badgers, respectively.

RABV phylogeny was analyzed by maximum likelihood (ML) and Bayesian methods for each N, M, and G genes to ensure the consistency of tree topology. Both methods showed a similar tree topology, and all of the RABV isolates from Taiwan formed a monophyletic group and can be further divided into three sub-clusters. Sub-cluster I, II, and III were isolated from Eastern, Central and Southern Taiwan, respectively (Figure 1).

All of the nucleotide substitution rates and times of most recent common ancestor (tMRCA) are summarized in Table 1. The Markov-chain Monte Carlo (MCMC) results showed that the relaxed clock model was a significantly better fit model than the strict clock model for each of the datasets. The uncorrelated exponential clock model was better fit for G and M genes, while the uncorrelated lognormal was better for the N gene. The estimated nucleotide substitution rates for the N gene was 2.49 × 10^−4^ substitutions/site/year (95% highest posterior density (HPD): 1.40 × 10^−4^–3.63 × 10^−4^), for the M gene was 4.03 × 10^−4^ substitutions/site/year (95% HPD: 1.63 × 10^−4^–6.55 × 10^−4^), and for the G gene was 4.75 × 10^−4^ substitutions/site/year (95% HPD: 2.05 × 10^−4^–7.32 × 10^−4^) (Table 1).

The tMRCA for the global RABV and ferret badger rabies virus were estimated based on different parts of the RABV gene. The tMRCA was estimated at 523–721 years for global RABV and 306–428 years in the Chinese ferret badger lineage (Table 1). The tMRCA of the Formosan ferret badger was estimated at 170 years (95% HPD between 83 and 270), 89 years (95% HPD between 32 and 166), and 75 years (95% HPD between 27 and 155) based on N, M, and G genes respectively (Table 1). In addition, the evolutionary rate of synonymous position (third codon position) was significantly higher than that of non-synonymous position (first and second codon position) in N, M, and G genes (Table 1).

### 2.2. Selection Pressures and Co-Evolution RABV in the Formosan Ferret Badger

The selection pressures on N, M, and G proteins were estimated on the dN/dS ratio. The criteria were (1) the ratio of dN/dS < 1 as negative selection; (2) dN/dS = 1 as neutrality; and (3) dN/dS > 1 as positive selection [12,13]. The mean ratio of dN/dS for N, M, and G proteins was 0.038, 0.080, and 0.112, respectively. No positive selection site has been detected on these genes, nor has a co-evolution relationship between N, M, and G proteins been found.

### 2.3. Phylogeographic of RABV in the Formosan Ferret Badger

**Figure 2:**
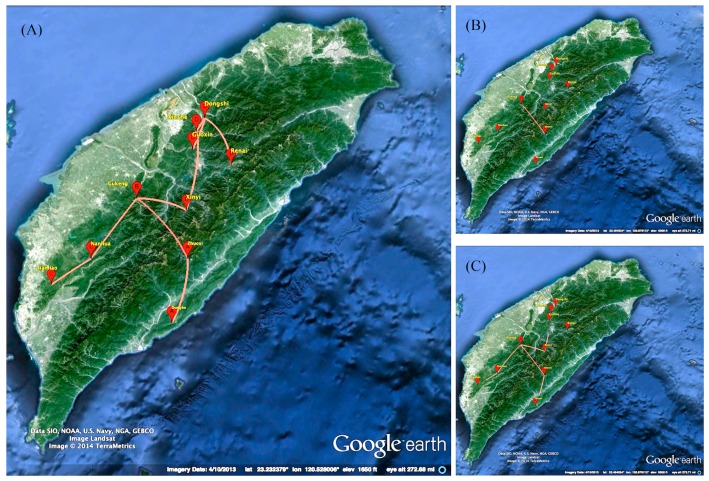
The migrations of temporal dynamics for the rabies virus in the Formosan ferret badger. (**A**) The branches expressed an overview of the spread in Taiwan; (**B**) The first stage was spreading from east to the west side; and (**C**) The second stage was spreading over into two branches and moved to southern and central regions. Map data: Google earth and TerraMetrics.

All of the three genes have a consistent result in phylogeography (Figure 2), indicating the possible transmission route of RABV in the Formosan ferret badger. The virus was latent in Eastern Taiwan, then crossed the Central Range to the western side of Taiwan and, subsequently, two branches of the virus separated along the mountainous area and conceal in the Formosan ferret badger (Figure 2).

## 3. Discussion

Owing to the good quarantine policy for animal importation, Taiwan has been a rabies-free country for at least 50 years. Surprisingly, the rabies virus was found in a dead Formosan ferret badger in July 2013, and then followed by an outbreak in this animal species in many regions of Taiwan, except the northern part. Surveillance data from government indicated the rabies cases are still ongoing and limited in the Formosan ferret badger only. To prevent the spread of rabies, the current public health control measures require mandatory vaccinations for dogs and cats against rabies, high risk workers should receive rabies immunization, and avoid taking dogs or other susceptible animals into prevalent area to minimize the contact with wild animals.

Before this outbreak, badger-associated RABV were only found in southeast region of China (Jiangxi and Zhejiang provinces) [14,15]. Therefore, the correlation of RABV genome evolution between Taiwan and Chinese ferret badgers is interesting to investigate. However, they have no direct evolutionary relationship according to the phylogenetic trees (Figure 1). The Chinese badger-associated RABV has formed an independent lineage, and was distinct from the RABV in dogs in the same regions.

The nucleotide substitution rates for the N, M, and G genes, were similar to that of previous reports in China, North America, Africa, and the other places [6,16,17,18,19]. In addition, the selection pressures and co-evolution analysis showed a significantly low mean of dN/dS (0.038–0.112) which is in accordance with other studies [5,16,18,19,20]. This suggested that the evolutionary pressure acting on this virus was strong purifying selection. No positive selection site and no evidence for co-evolution have been found in RABV isolated from the Formosan ferret badger. This observation reflects the fact that Taiwan was a rabies-free country for a long time; therefore, there was no need need for any vaccination program for wild animals and, thus, RABV was free from selection pressure.

**Figure 3:**
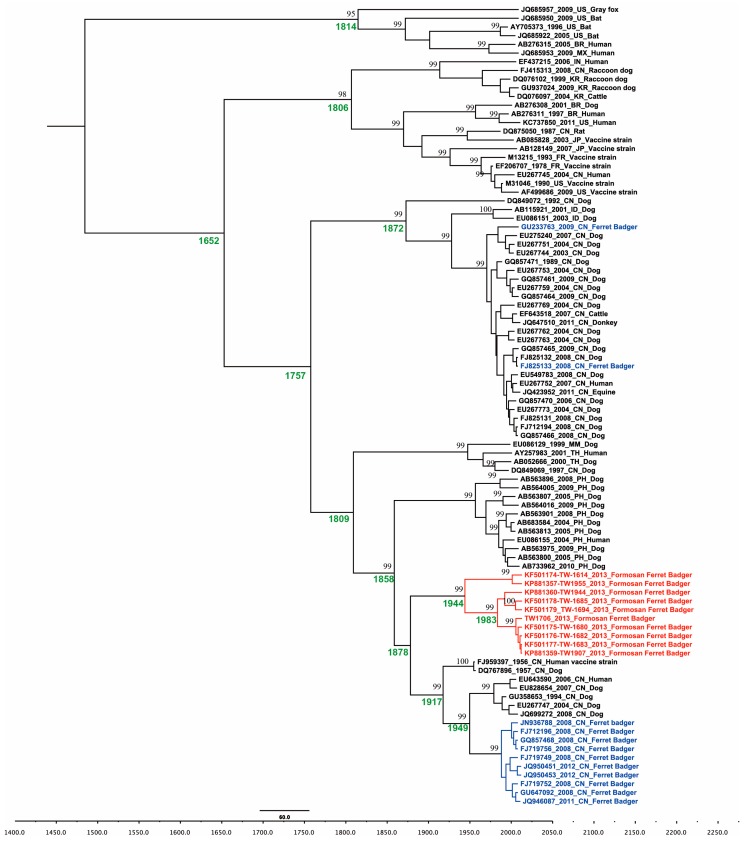
The Maximum Clade Credibility (MCC) tree of the rabies virus based on glycolprotein. The MCC tree was constructed with 10% burn-in by Tree Annotator v 1.8 implemented in the BEAST software package. Numbers beside the branches are posterior probability and branching time (Text in green). Only posterior probability values above 0.95 are shown. Texts in red and blue represent the RABV isolated from Formosan and Chinese ferret badgers, respectively.

The data of tMRCA estimated from our study suggested RABV was latent in Taiwan mountainous areas for at least 70 years (Figure 3). Phylogeographic analysis showed the transmission of RABV in Formosan ferret badgers likely originated from Eastern Taiwan, then moved across the Central Range to western regions (Figure 2). Subsequently, the virus was separated into two lineages and moved to central and southern regions. Interestingly, Northern Taiwan remains a rabies-free area bordered by the Da-An River as a natural barrier. Nevertheless, Formosan ferret badgers are widely distributed on the whole island, including Northern Taiwan and the Da-An River seems unlikely to be a difficult natural barrier to cross for the animals. The reason why RABV did not move forward to Northern Taiwan remains unclear. The origin of RABV in Formosan ferret badgers is another ambiguous question.

In conclusion, this study investigated the phylogeographic and molecular evolution of the re-emergence of RABV in the Formosan ferret badger. Our results showed this virus has evolved independently in Taiwan for a long time and their phylogenetic lineages were evolved from geographic segregation across the Central Range from the eastern to the western side.

## 4. Materials and Methods

### 4.1. Source of Sequences

All the analyzed RABV sequences from infected Formosan ferret badgers were authorized and obtained from Animal Health Research Institute, Council of Agriculture, Executive Yuan, Taiwan. These included full-length of eight nucleoprotein genes (1353 bp), 10 matrix proteins (609 bp), and 10 glycoproteins (1575 bp) from this outbreak, and another 71 N, 39 M, and 82 G gene sequences retrieved from the NCBI GenBank database, including Asia, Europe, America, and Africa, as the reference for molecular evolution analysis. The accession numbers of sequences used for phylogenetic and evolutionary analysis in this study were listed in supplementary table (Table S1).

### 4.2. Phylogenetic Analysis

Full lengths of each segment genes were aligned using the T-Coffee method [21]. The transition/transversion ratio, gamma distribution rates, and base frequency were estimated by the TREE-PUZZLE software version 5.2 [22]. To ensure the consistency of the tree topology, phylogenetic trees were constructed using the maximum likelihood (ML) and Bayesian method in PhyML 3.0 and BEAST v1.8 respectively [23,24]. Branch support was evaluated by bootstrap analysis based on 1000 replicates for the ML tree. The bootstrap value >75% was considered to a monophyletic group.

### 4.3. Phylodynamic and Phylogeography Analysis

To analyze the evolutionary rate of RABV in Taiwan, we applied the Bayesian Markov Chain Monte Carlo (MCMC) method offered in the BEAST software v1.8.2 (Available online: http://beast.bio.ed.ac.uk/) along with the BEAGLE library [25]. The Shapiro-Rambaut-Drummond-2006 (SRD06) substitution model was used in the BEAST software for each gene region because this model was recognized to provide better resolution for coding regions in Bayesian analysis [26]. The strict, exponential-relaxed and lognormal-relaxed molecular clock models with constant size coalescent was tested to estimate the evolution pattern [27]. The best fit demographic and clock model was estimated from a model comparison by Akaike’s information criterion (AICM) in the Tracer program v1.6 [28]. The MCMC chains were run for sufficient time to achieve convergence (Effective Sample Size (ESS) > 200). In addition, the uncertainty of parameters were estimated in the 95% highest probability density (HPD) region. The Maximum Clade Credibility (MCC) tree was constructed by Tree Annotator v 1.8 (Included in the BEAST software package), with 10% burn-in and then edited by FigTree v1.4.2 (Available online: http://tree.bio.ed.ac.uk/).

The phylogeographic analysis was performed in BEAST V1.8.2 [27]. A discrete trait substitution model was the symmetric substitution model with Bayesian stochastic search variable selection (BSSVS). The MCC tree was converted to a keyhole markup language file (KML file) using SPREAD [29] and imported to Google Earth (Available online: http://www.google.com/earth) to produce a graphical animation of the estimated spatio-temporal movements of RABV lineages in Formosan ferret badgers.

### 4.4. Selection Pressure and Co-Evolution Analysis

To determine the selection pressures on N, M, and G genes, we estimated the ratio of non-synonymous substitutions (dN) and synonymous substitutions (dS) per site based on ML trees under the appropriate substitution model, using the single-likelihood ancestor counting (SLAC), fixed effects likelihood (FEL), and internal fixed effects likelihood (IFEL) methods with significance level *p*-values of 0.05. All analysis was performed on the Datamonkey website interface (Available online: http://www.datamonkey.org) [12,30]. Furthermore, we used Spidermonkey to reconstruct the substitution history of N, M, and G by ML-based methods, and analyzed the joint distribution of substitution events by Bayesian graphical models (BGMs) to identify significant associations among sites [31].

## Figures and Tables

**Table 1:** Mean relative evolutionary rates for codon positions and times of most recent common ancestor (tMRCA) in nucleoprotein, matrix protein, and glycoprotein genes.

Gene	tMRCA	Substitution Rates Sub/Site/Year (10^−4^)	Mean Relative Substitution Rate	Standard Error of Mean
Nucleoprotein	721.62 (406.06–1096.16)	2.49 (1.40–3.63)	-	-
Ferret badger (Taiwan)	170.37 (83.22–270.70)	-	-	-
Ferret badger (China)	428.67 (243.95–644.24)	-	-	-
1st + 2nd codon position	-	-	0.22 (0.19–0.26)	1.26 × 10^−4^
3rd codon position	-	-	2.55 (2.48–2.62)	2.52 × 10^−4^
Matrix protein	523.03 (218.04–931.53)	4.03 (1.63–6.55)	-	-
Ferret badger (Taiwan)	89.55 (32.37–166.28)	-	-	-
Ferret badger (China)	391.62 (173.13–692.32)	-	-	-
1st + 2nd codon position	-	-	0.30 (0.24–0.36)	2.05 × 10^−4^
3rd codon position	-	-	2.395 (2.28–2.51)	4.09 × 10^−4^
Glycoprotein	630.69 (206.44–1309.38)	4.75 (2.05–7.32)	-	-
Ferret badger (Taiwan)	75.68 (26.03–136.00)	-	-	-
Ferret badger (China)	306.95 (105.09–578.58)	-	-	-
1st + 2nd codon position codon	-	-	0.37 (0.33–0.40)	1.37 × 10^−4^
3rd codon position	-	-	2.26 (2.19–2.34)	2.75 × 10^−4^

## Supplementary Materials

Supplementary materials can be found at http://www.mdpi.com/1422-0067/17/3/392/s1.
