# Supplementary material for: Spatial Temporal Dynamics and Molecular Evolution of Re-Emerging Rabies Virus in Taiwan
Source: Int J Mol Sci. 2016 Mar 17;17(3):392. doi: 10.3390/ijms17030392 (PMC4813248; doi:10.3390/ijms17030392)
Supplement: Supplementary file 1 [file ijms-17-00392-s001.pdf]

Supplementary Materials: Spatial Temporal Dynamics and Molecular Evolution of Re-Emerge Rabies Virus in Taiwan

Yung-Cheng Lin, Pei-Yu Chu, Mei-Yin Chang, Kuang-Liang Hsiao, Jih-Hui Lin and Hsin-Fu Liu

Table S1. The list of references strains used to phylogenetic and evolutionary analysis in this study.

Rabies Virus Glycoprotein Gene					
**Accession**	**Isolate**	**Country**	**Country Code**	**Host Species**	**Year**
AB683584	RV/NCR.PHL/2004/Z-04-522	Philippines	PH	Dog	2004
AB563813	RV/NCR.PHL/2005/Z-05-396	Philippines	PH	Dog	2005
AB563901	RV/R3.PHL/2008/TRa-086	Philippines	PH	Dog	2008
EU086155	04030PHI	Philippines	PH	Human	2004
AB563800	RV/NCR.PHL/2005/Z-05-009	Philippines	PH	Dog	2005
AB563975	RV/CAR.PHL/2009/TRa-200	Philippines	PH	Dog	2009
AB733962	RV/CAR.PHL/2010/TRa-289	Philippines	PH	Dog	2010
AB564005	RV/R7.PHL/2009/TRa-239	Philippines	PH	Dog	2009
AB563896	RV/R7.PHL/2008/TRa-080	Philippines	PH	Dog	2008
AB564016	RV/R10.PHL/2009/TRa-252	Philippines	PH	Dog	2009
AB563807	RV/R11.PHL/2005/Z-05-186	Philippines	PH	Dog	2005
FJ959397	CTN-1	China	CN	Vaccine	1956
DQ767896	CTN	China	CN	Dog	1957
JQ699272	CGX0810D	China	CN	Dog	2008
EU267747	Guizhou Al03	China	CN	Dog	2004
GU358653	GX4	China	CN	Dog	1994
EU828654	GN07	China	CN	Dog	2007
EU643590	HN10	China	CN	Human	2006
JN936788	CZJ0803F	China	CN	Ferret Badger	2008
FJ712196	F04	China	CN	Ferret Badger	2008
GQ857468	08ZL11	China	CN	Ferret Badger	2008
FJ719756	ZJ-LA	China	CN	Ferret Badger	2008
JQ946087	JX08-45CC	China	CN	Ferret Badger	2011
GU647092	JX08-45	China	CN	Ferret Badger	2008
FJ719752	JX08-48	China	CN	Ferret Badger	2008
JQ950453	JX12-234	China	CN	Ferret Badger	2012
FJ719749	JX08-47	China	CN	Ferret Badger	2008
JQ950451	JX12-102	China	CN	Ferret Badger	2012
EU086129	9913BIR	Myanmar	MM	Dog	1999
AY257983	HM208	Thailand	TH	Human	2001
DQ849069	N11	China	CN	Dog	1997
AB052666	No.33	Thailand	TH	Dog	2000
DQ849072	CQ92	China	CN	Dog	1992
EU086151	03003INDO	Indonesia	ID	Dog	2003
AB115921	SN01-23	Indonesia	ID	Dog	2001
EU267744	Guangxi_YL66	China	CN	Dog	2003
EU267751	Guizhou Qx5	China	CN	Dog	2004
EU275240	Yunnan_Qj07	China	CN	Dog	2007
GQ857464	09AF33	China	CN	Dog	2009
EU267759	Henan Sq59	China	CN	Dog	2004
GQ857461	09AF30	China	CN	Dog	2009
EU267753	Henan Hb10	China	CN	Dog	2004
GQ857471	WJ	China	CN	Dog	1989
EU267769	HunanXx33	China	CN	Dog	2004
JQ647510	WH11	China	CN	Donkey	2011
EF643518	Hubei/Wuhan/070308	China	CN	Buffalo	2007
EU267762	Hunan DK13	China	CN	Dog	2004
EU267763	Hunan Wg12	China	CN	Dog	2004
GU233763	JX09-17(fb)	China	CN	Ferret Badger	2009
FJ825132	D11	China	CN	Dog	2008
FJ825133	F01	China	CN	Ferret Badger	2008

GQ857465	ZJCA1	China	CN	Dog	2009
FJ712194	D02	China	CN	Dog	2008
GQ857466	08ZL01	China	CN	Dog	2008
FJ825131	D10	China	CN	Dog	2008
GQ857470	LH	China	CN	Dog	2006
EU267773	Jiangsu Wx1	China	CN	Dog	2004
EU267752	Hebi0(H)	China	CN	Human	2007
JQ423952	BJ2011E	China	CN	Equine	2011
EU549783	BD06	China	CN	Dog	2008
DQ076097	SKRBV0404HC	Korea	KR	Cattle	2004
GU937024	KRVR0906	Korea	KR	Raccoon dog	2009
DQ076102	SKRRD9902PJ	Korea	KR	Raccoon dog	1999
FJ415313	NeiMeng925	China	CN	Raccoon dog	2008
EF437215	NNV-RAB-H	India	IN	Human	2006
KC737850	A11-5300	USA	US	Human	2011
AB276311	BRhm528	Brazil	BR	Human	1997
AB276308	BRdg101	Brazil	BR	Dog	2001
AB085828	HEP-Flury	Japan	JP	Vaccine	2003
DQ875050	MRV	China	CN	Rat	1987
AB128149	Ni-CE	Japan	JP	Vaccine	2007
M13215	PV	France	FR	Vaccine	1993
EF206707	ERV	USA	US	Vaccine	1978
M31046	SAD B19	USA	US	Vaccine	1990
EU267745	Guizhou Al0(H)	China	CN	Human	2004
AF499686	SRV9	China	CN	Vaccine	2006
JQ685957	OR8767	USA	US	Gray fox	2009
JQ685922	TN186	USA	US	Bat	2005
AY705373	SHBRV-18	USA	US	Bat	1996
JQ685950	A093504	USA	US	Bat	2009
JQ685953	3645DR	Mexico	MX	Human	2009
AB276315	BRhm671	Brazil	BR	Human	2005
KF501174	TW-1614	Taiwan	TW	Formosan Ferret Badger	2013
KF501175	TW-1680	Taiwan	TW	Formosan Ferret Badger	2013
KF501176	TW-1682	Taiwan	TW	Formosan Ferret Badger	2013
KF501177	TW-1683	Taiwan	TW	Formosan Ferret Badger	2013
KF501178	TW-1685	Taiwan	TW	Formosan Ferret Badger	2013
KF501179	TW-1694	Taiwan	TW	Formosan Ferret Badger	2013
N/A	TW-1706	Taiwan	TW	Formosan Ferret Badger	2013
KP881359	TW-1907	Taiwan	TW	Formosan Ferret Badger	2013
KP881360	TW-1944	Taiwan	TW	Formosan Ferret Badger	2013
KP881357	TW-1955	Taiwan	TW	Formosan Ferret Badger	2013
**Rabies Virus Nucleoprotein Gene**					
**Accession**	**Isolate**	**Country**	**Country Code**	**Host Species**	**Year**
HM486368	CGZ0508D	China	CN	Dog	2005
DQ666290	Guizhou_A103	China	CN	Dog	2004
DQ866121	GXWXp	China	CN	Dog	2006
EU159398	nu	China	CN	Human	1986
EU086182	02046CHI	China	CN	Dog	1994
EU159386	GX4	China	CN	Dog	1994
DQ866105	GX01	China	CN	Dog	2006
DQ866116	GXLA	China	CN	Dog	2003
DQ866120	GXSL	China	CN	Dog	2004
EU643590	HN10	China	CN	Human	2006
EU828653	GN07	China	CN	Dog	2007
FJ712196	F04	China	CN	Ferret Badger	2008
FJ598135	ZJ-LA	China	CN	Ferret Badger	2008
JQ946087	JX08-45CC	China	CN	Ferret Badger	2011
FJ719753	Jx08-48	China	CN	Ferret Badger	2008
GU647092	JX08-45	China	CN	Ferret Badger	2008
FJ719751	Jx08-47	China	CN	Ferret Badger	2008
JQ950452	Jx12-234	China	CN	Ferret Badger	2012
JQ950448	JX12-67	China	CN	Ferret Badger	2012
FJ959397	CTN-1	China	CN	Vaccine	1956

HM486380	CSD0614D	China	CN	Dog	2006
DQ787145	CTN	China	CN	Vaccine	1957
EU086205	04030PHI	Philippines	PH	Dog	2004
AY103004	94273PHI	Philippines	PH	Human	1994
EU275243	Yunnan_Tc06	China	CN	Dog	2006
FJ594278	N11	China	CN	Dog	1997
JN786877	QS-05	Thailand	TH	Dog	2012
EU086206	8734THA	Thailand	TH	Human	1983
EU086171	9916CBG	Cambodia	KH	Dog	1999
EU086209	01016VNM	Viet Nam	VN	Dog	2001
EU086210	01017VNM	Viet Nam	VN	Dog	2001
EU086192	03003INDO	Indonesia	ID	Dog	2003
EU275245	Yunnan_Qj07	China	CN	Dog	2007
EU159401	WJ	China	CN	Dog	1989
HQ118110	ZJD8	China	CN	Dog	2008
JQ647510	WH11	China	CN	Donkey	2011
EF611081	hubei070308	China	CN	Buffalo	2007
GU233765	JX09-17-FB	China	CN	Ferret Badger	2009
EU828657	WJ07-1	China	CN	Dog	2007
EU549783	BD06	China	CN	Dog	2008
EU828655	GC07	China	CN	Dog	2007
EU267777	Hebei0(H)	China	CN	Human	2007
EF556197	Zhejiang Wz0(H)	China	CN	Human	2006
JQ685957	OR8767	USA	US	Gray fox	2009
GU644982	TN186	USA	US	Bat	2005
AY705373	SHBRV-18	USA	US	Bat	1996
JQ685950	A093504	USA	US	Bat	2009
AF351849	STTEX	USA	US	Bat	1994
JQ685953	3645DR	Mexico	MX	Human	2009
KC758862	ARGA795_2007	USA	US	Bat	2007
AY877435	V920	Mexico	MX	Bat	1993
GU937036	KRVR0901	Korea	KR	Raccoon dog	2009
DQ076130	SKRBV0404HC	Korea	KR	Cattle	2004
DQ076124	SKRDG0203CW	Korea	KR	Dog	2002
EF437215	NNV-RAB-H	India	IN	Human	2006
AY352493	RV61	India	IN	Human	1987
L20672	Ontario	Canada	CA	Ontario fox	1996
AY352486	3510w	Russian	RU	Wolf	1995
AY352462	RVHK	Russian	RU	Human	1998
EU853594	Guinea	Guinea	GN	Dog	1993
AY854593	V684	Mexico	MX	skunk	1992
AB128149	Ni-CE	Japan	JP	Vaccine	2007
AB044824	Nishigahara	Japan	JP	Vaccine	2000
EF206715	SAD P5/88(Rabifox)	USA	US	Vaccine	2001
AF499686		USA	US	Vaccine	2006
EF206707	ERA	France	FR	Vaccine	1978
AF357308	PV	France	FR	Vaccine	1993
AB085828	HPE-Flury	Japan	JP	Vaccine	2003
DQ875050	MRV	China	CN	Rat	1987
KF501180	TW-1614	Taiwan	TW	Formosan Ferret Badger	2013
KF501181	TW-1680	Taiwan	TW	Formosan Ferret Badger	2013
KF501182	TW-1682	Taiwan	TW	Formosan Ferret Badger	2013
KF501183	TW-1683	Taiwan	TW	Formosan Ferret Badger	2013
KF501184	TW-1685	Taiwan	TW	Formosan Ferret Badger	2013
KF501185	TW-1694	Taiwan	TW	Formosan Ferret Badger	2013
N/A	TW-1749	Taiwan	TW	Formosan Ferret Badger	2013
KP881356	TW-1944	Taiwan	TW	Formosan Ferret Badger	2013
**Rabies Virus Matrix Protein Gene**					
**Accession**	**Isolate**	**Country**	**Country Code**	**Host Species**	**Year**
GU358653	GX4	China	CN	Dog	1994
GQ472533	GXWX	China	CN	Pig	2005
GQ472507	GX01	China	CN	Dog	2004
EU643590	HN10	China	CN	Human	2006

FJ959397	CTN-1	China	CN	Vaccine	1956
FJ712195	F02	China	CN	Ferret Badger	2008
FJ719757	ZJ-LA	China	CN	Ferret Badger	2008
FJ719754	JX08-58	China	CN	Ferret Badger	2008
GU647092	JX08-45	China	CN	Ferret Badger	2008
JQ946087	JX08-45CC	China	CN	Ferret Badger	2011
FJ719750	JX08-47	China	CN	Ferret Badger	2008
JQ647510	WH11	China	CN	Donkey	2011
GU233764	JX09-17(fb)	China	CN	Ferret Badger	2009
FJ719759	ZJ-QZ	China	CN	Dog	2008
EU549783	BD06	China	CN	Dog	2008
FJ032326	D02	China	CN	Dog	2008
EU293100	8764THA	Thailand	TH	Human	1983
EU293107	8743THA	Thailand	TH	Human	1983
JN786877	QS-05	Thailand	TH	Dog	2012
AB699220	BDR5	Bangladesh	BD	Goat	2010
KF154996	RV61	India	IN	Human	1987
EF437215	NNV-RAB-H	India	IN	Human	2006
KC171644	BD0406CC	Korea	KR	Raccoon dog	2004
KC171645	BV9901PJ	Korea	KR	Raccoon dog	1999
AB291107	BR-Pfx6	Brazil	BR	Gray fox	2002
AB085828	HPE-Flury	Japan	JP	Vaccine	2003
DQ875050	MRV	China	CN	Rat	1987
JQ977406	1352KRA	Russian	RU	Dog	2008
EU307994	CVS	India	IN	Dog	1988
EF206707	ERA	France	FR	Vaccine	1978
EF206715	SAD P5/88 (Rabifox)	USA	US	Vaccine	2001
FJ032330	F01	China	CN	Ferret Badger	2008
AB128149	Ni-CE	Japan	JP	Vaccine	2007
AB044824	Nishigahara	Japan	JP	Vaccine	2000
AF360857	NYRAC; NY516	USA	US	Procyon lotor	1997
JQ685953	3645DR	Mexico	MX	Human	2009
JQ685957	OR8767	USA	US	Gray Fox	2009
JQ685950	A093504	USA	US	Bat	2009
JQ685922	TN186	USA	US	Bat	2005
KF544960	TW-1614	Taiwan	TW	Formosan Ferret Badger	2013
KF544961	TW-1680	Taiwan	TW	Formosan Ferret Badger	2013
KF544962	TW-1682	Taiwan	TW	Formosan Ferret Badger	2013
KF544963	TW-1683	Taiwan	TW	Formosan Ferret Badger	2013
KF544964	TW-1685	Taiwan	TW	Formosan Ferret Badger	2013
KF544965	TW-1694	Taiwan	TW	Formosan Ferret Badger	2013
N/A	TW-1706	Taiwan	TW	Formosan Ferret Badger	2013
N/A	TW-1749	Taiwan	TW	Formosan Ferret Badger	2013
N/A	TW-1879	Taiwan	TW	Formosan Ferret Badger	2013
N/A	TW-1944	Taiwan	TW	Formosan Ferret Badger	2013

Abbreviations: BD: Bangladesh; BR: Brazil; CA: Canada; CN: China; FR: France; GN: Guinea; ID: Indonesia; IN: India; JP: Japan; KH: Cambodia; KR: Korea; MM: Myanmar; MX: Mexico; PH: Philippines; RU: Russian; TH: Thailand; TW: Taiwan; US: United States of America (USA); VN: Viet Nam.
